# Renewal and reacquisition of substance-associated relapse

**DOI:** 10.3389/fpsyg.2026.1781712

**Published:** 2026-05-13

**Authors:** Isabelle Rodríguez-Lausell, Tyler M. Goold, Roberto G. Fileni, Ragini Aggarwal-Matto, Freddyson J. Martínez-Rivera

**Affiliations:** Department of Neuroscience, McKnight Brain Institute, College of Medicine, Center for Addiction Research and Education, University of Florida, Gainesville, FL, United States

**Keywords:** addiction, drug, extinction, reinstatement, reward, relapse

## Abstract

Renewal and reacquisition (R/R) of drug-seeking behaviors are key drivers of relapse in substance use disorders (SUDs). These processes emerge from interactions among learning, memory, and reward-related neural circuits that are engaged when individuals encounter drug-associated contexts, cues, stressors, or restored drug availability. Advances in animal models and experimental tools have improved our understanding of the neurobiological mechanisms that cause relapse, including how substance-associated memories are formed and retrieved, how context-dependent renewal happens after extinction or punishment-based interventions, and how substance seeking and taking restarts rapidly when reinforcement is restored. In this review, we synthesize evidence on behavioral, circuit-level and molecular processes that contribute to R/R across substances, highlighting translational and clinical parallels, and identifying mechanistic gaps that constrain intervention development. We conclude by outlining mechanism-informed strategies that integrate behavioral, pharmacological, and genetic interventions to strengthen the generalization (transference) and durability of extinction learning and memory updating, to reduce relapse vulnerability, particularly driven by R/R.

## 1 Introduction

**Figure 1:**
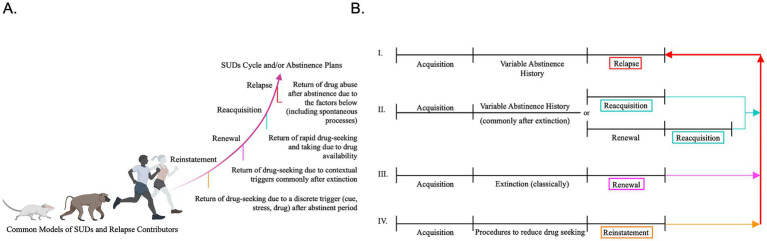
Conceptual overview of relapse-related behavioral models in substance use disorder research. **(A)** Schematic illustrating commonly used behavioral models of substance use disorders (SUDs) and relapse contributors across species, highlighting distinctions among reinstatement (e.g., after extinction or punishment strategies), renewal, reacquisition, and relapse based on the conditions under which drug seeking re-emerges following abstinence. Definitions emphasize differences in triggering factors, including discrete cues or stressors, contextual changes, drug availability, and spontaneous processes following abstinence. The curved trajectory is intended to represent a conceptual progression in sequence, complexity, and stimulus specificity across relapse-related processes that may contribute to relapse and the maladaptive cycle of SUD, rather than increasing severity (generated using Biorender). **(B)** Timeline representations of experimental procedures used to model relapse-related behaviors. Panels depict acquisition followed by distinct abstinence histories (e.g., extinction or variable abstinence) and subsequent tests for relapse, reacquisition, renewal, or reinstatement (e.g., after extinction or punishment strategies). Together, these schematics illustrate how differences in abstinence conditions and testing contexts operationally distinguish relapse models commonly used in the literature.

Relapse remains a major obstacle in the treatment of substance use disorders (SUDs), often occurring despite prolonged periods of abstinence. Substance-seeking and -taking behaviors can reemerge in response to substance-associated cues, contexts, or stressors, including conflicts and withdrawal-related states. Two critical processes that contribute to relapse are the renewal and reacquisition (R/R) of substance-associated memories that drive the resumption of substance use (Figure 1A) (Bouton, 2002; Crombag and Shaham, 2002; McNally, 2014). *Renewal:* is the return of an extinguished conditioned or instrumental response when tested outside the extinction context (rather than after a post-extinction delay in the same context, as in spontaneous recovery) (Bouton et al., 2012). In many preclinical models, abstinence is supported by extinction-related processes, in which drug omission or unavailability promotes inhibitory learning that suppresses drug seeking (Bouton, 2002). Extinction produces a new, context-dependent inhibitory memory that competes with the original drug-reward association. Preclinically, renewal is commonly measured using ABA designs, where drug self-administration is induced in Context A, extinguished in Context B, and then assessed following re-exposure to Context A (Crombag et al., 2008; Fuchs et al., 2006; McNally, 2014). This framework resembles clinical patterns of relapse after treatment (e.g., extinction-based approaches) or forced abstinence (including withdrawal) when individuals return to environments previously associated with drug use and renew the drug-associated memories (Bouton, 2002; Todd et al., 2014; Khoo et al., 2017). Consistent with this translational relevance, drug-associated renewal engages a distributed network of reward- and memory-related brain regions, including prefrontal, amygdalar, hippocampal, and striatal circuits, across multiple drug classes (Fuchs et al., 2004, 2005; Adhikary et al., 2017). *Reacquisition:* refers to the rapid return and resumption of drug-taking behavior when the original response–reinforcer contingency is restored. Often studied following explicit extinction training and renewal, though it can also occur after periods of abstinence without extinction, reflecting the persistence of excitatory drug memories despite abstinence or inhibitory learning (Bouton, 2002; Willcocks and McNally, 2011). Reacquisition is typically faster than initial acquisition, as latent substance–reward associations allow re-exposure to the reinforcer to bypass extinction-related inhibition, and it can be modulated independently of initial learning, implicating partially distinct neural and molecular mechanisms (Grimm et al., 2001; Willcocks and McNally, 2014).

Given their close conceptual and temporal relationship, where renewal often precedes and facilitates reacquisition, we refer to these processes collectively as R/R throughout this review. Behavioral paradigms used to study these and related relapse-like phenomena, including reinstatement and R/R, are summarized in Figures 1A,B, which highlights key procedural differences in how drug seeking reemerges across models. These R/R processes can operate independently of classic reinstatement paradigms, such as drug-priming or stress-induced relapse, and capture clinically common relapse trajectories that occur when individuals return to drug-associated environments and/or regain access to the drug (Everitt and Robbins, 2016; Kalivas and O’Brien, 2008). Because ‘reinstatement’ has historically been used broadly to encompass drug-, cue-, stress-, and sometimes context-induced relapse, the term is often applied without explicit consideration of extinction history or contextual shifts, which are central to the concept of renewal. Thus, renewal refers more specifically to relapse driven by contextual shifts following extinction, particularly when responding reemerges outside the extinction context, thereby emphasizing the context dependence of extinction memories. Notably, R/R shares core mechanisms with other relapsing scenarios and conditions (e.g., anxiety disorders), in which extinction or abstinence does not erase original memories but instead leaves them context dependent and susceptible to rapid behavioral re-engagement upon re-exposure (reacquisition) (Bouton, 2002; Todd et al., 2014) (Figure 1A).

In this review, we synthesize evidence on R/R across behavioral, circuitry, molecular and translational domains, with primary emphasis on instrumental self-administration paradigms implementing the ABA design. Our literature search was inclusive of all substance classes; however, the available R/R literature was concentrated within stimulants, depressants, opioids, nicotine, and cannabinoids (through Pubmed search string). Overall, the evidence reviewed here is primarily rodent-based, with most studies conducted in rats and mice, and when available, we explicitly indicate the substance, species, and sex represented in the studies discussed. We also note that most preclinical studies were conducted in males unless otherwise specified, underscoring the limited consideration of sex as a biological variable in the current literature.

**Figure 2:**
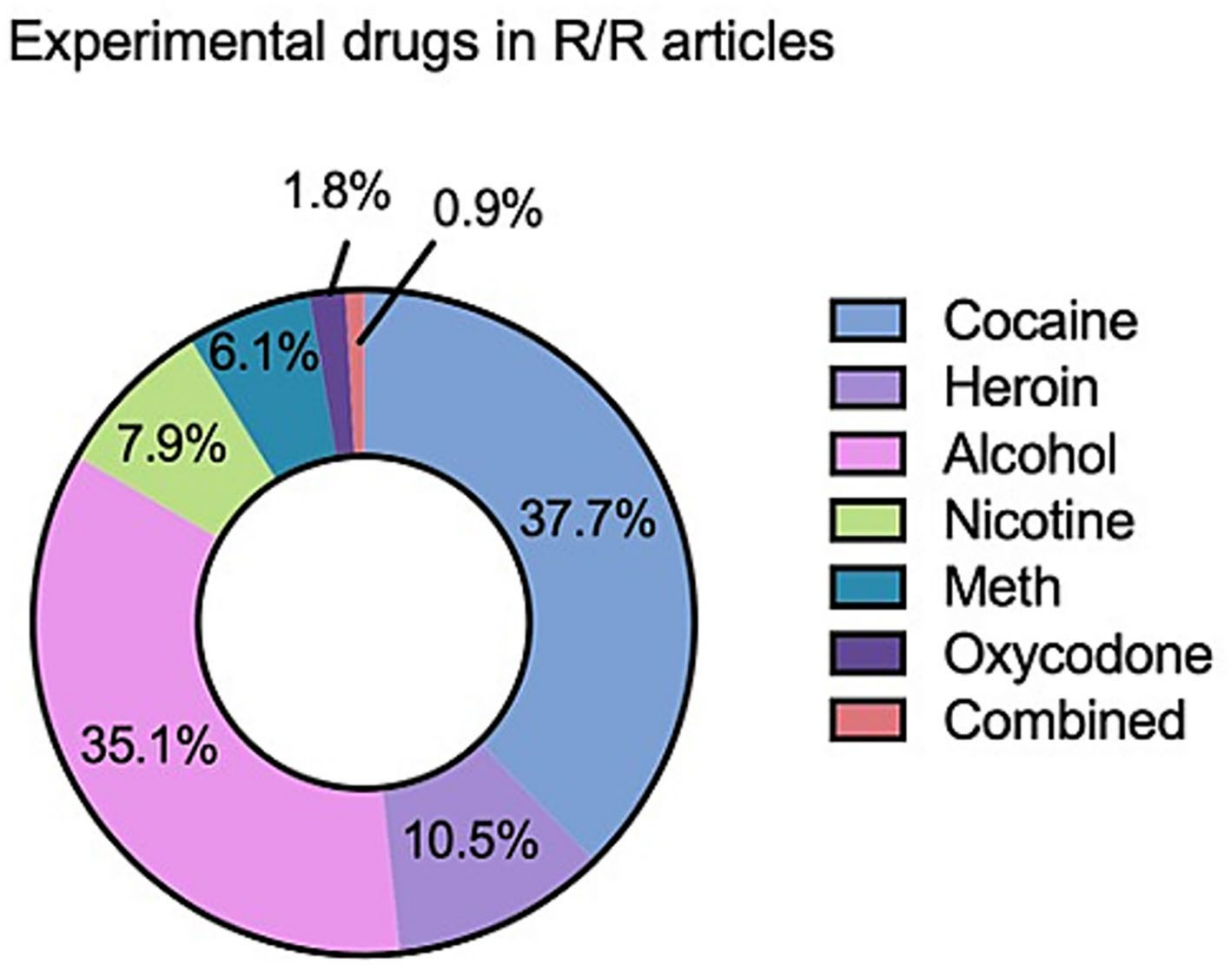
Analysis of papers meeting the inclusion criteria. Donut chart depicting the proportional representation of drug classes examined in articles that met the inclusion criteria for the present review (*N* = 114). Sections reflect the number of studies investigating each drug class relative to the total number of included articles. Generated using Prism version 10. The inclusion criteria are: (“renewal”[Title/Abstract] OR “reacquisition”[Title/Abstract] OR “relapse”[Title/Abstract]). AND (“drug”[Title/Abstract] OR “cocaine”[Title/Abstract] OR “heroin”[Title/Abstract] OR “fentanyl”[Title/Abstract] OR “benzodiazepine*”[Title/Abstract] OR “THC”[Title/Abstract] OR “cannabis”[Title/Abstract] OR “marijuana”[Title/Abstract] OR “nicotine”[Title/Abstract] OR “alcohol”[Title/Abstract] OR “ethanol”[Title/Abstract] OR “methamphetamine”[Title/Abstract] OR “amphetamine*”[Title/Abstract] OR “psychostimulant*”[Title/Abstract])). AND (“rat”[Title/Abstract] OR “rats”[Title/Abstract] OR “mouse”[Title/Abstract] OR “mice”[Title/Abstract] OR “rodent*”[Title/Abstract] OR “human”[Title/Abstract] OR “humans”[Title/Abstract] OR “nonhuman primate*”[Title/Abstract] OR “monkey”[Title/Abstract] OR “macaque”[Title/Abstract]). AND (“self-administration”[All Fields] OR “pavlovian learning”[All Fields] OR “operant conditioning”[All Fields] OR “extinction”[All Fields] OR “extinction learning”[All Fields] OR “ABA”[All Fields] OR “ABB”[All Fields])). NOT (review[pt] OR systematic[sb] OR meta-analysis[pt] OR comment[pt] OR editorial[pt] OR letter[pt] OR congresses[pt]). NOT (“fear”[Title] OR “anxiety”[Title] OR “stress”[Title] OR “ptsd”[Title] OR “trauma”[Title]).

**Figure 3:**
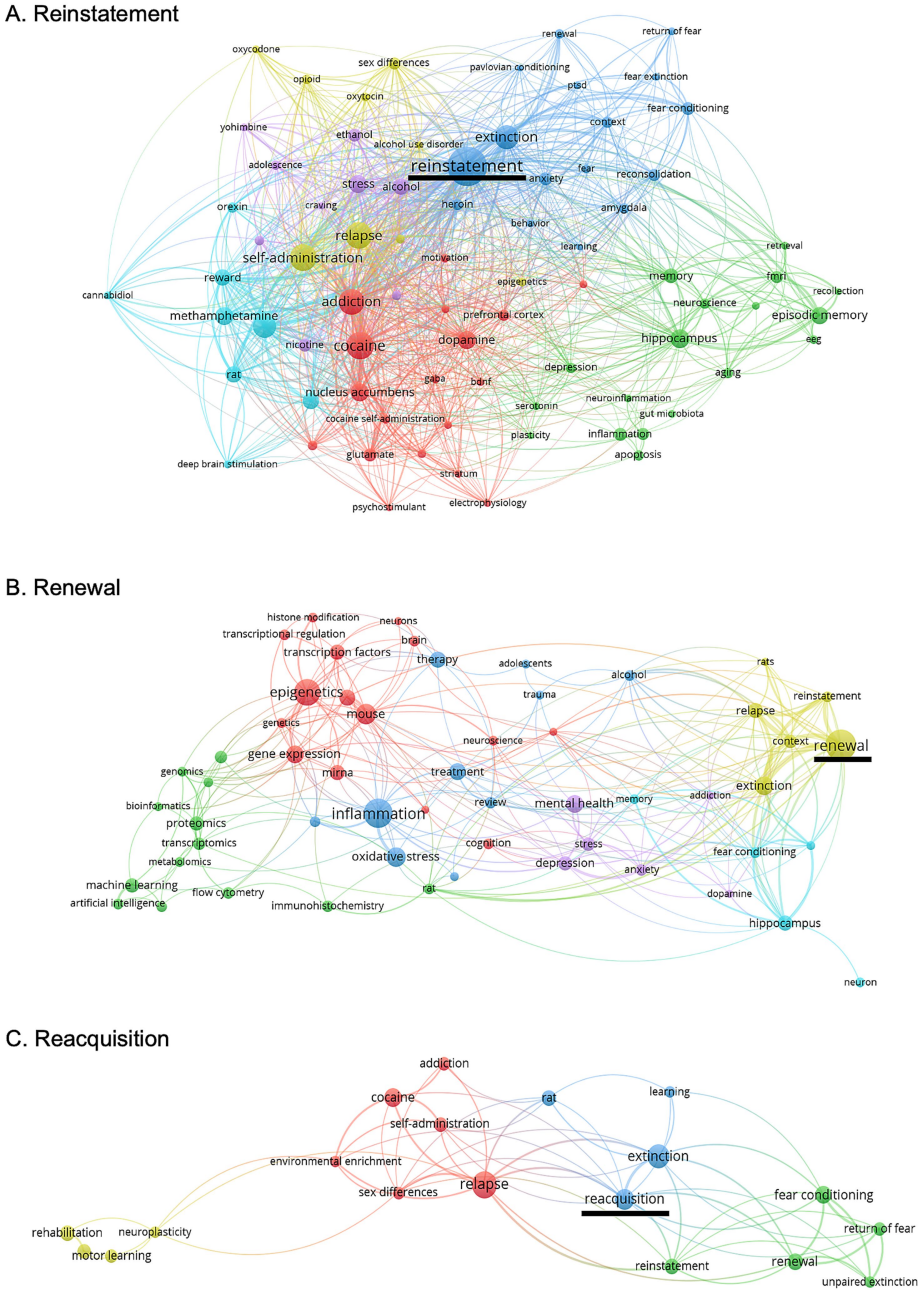
Analysis of articles published from 1986 to 2025 on relapse modalities. **(A)** Hot topics on reinstatement, **(B)** renewal, and **(C)** reacquisition. Generated using VOS viewer.

**Figure 4:**
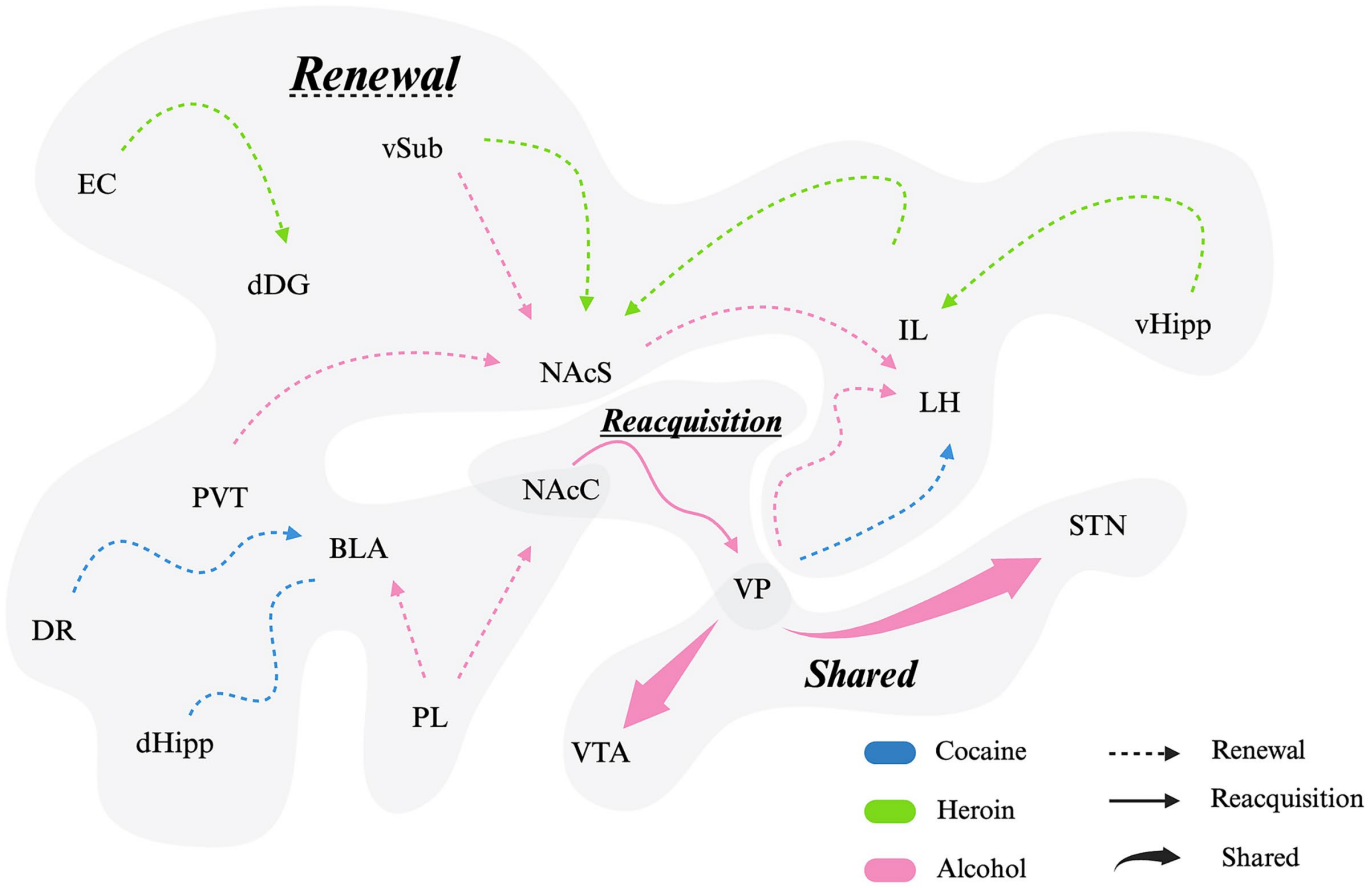
Main distinct and overlapping circuits between renewal and reacquisition. Dashed arrows/lines (−--) represent renewal circuitry, solid arrows/lines (—) denote reacquisition circuitry, and thick arrows/lines (**—**) highlight pathways shared between renewal and reacquisition. Blue indicates cocaine-associated circuits, pink represents alcohol-related circuits, and green denotes heroin-associated circuits. Word size (‘Renewal’, ‘Reacquisition’, and ‘Shared’) represents the proportion of circuits (literature) found. BLA = basolateral amygdala, DR = dorsal raphe, EC = entorhinal cortex, dDG = dorsal dentate gyrus (hippocampus), dHipp = dorsal hippocampus, vHipp = ventral hippocampus, vSub = ventral subiculum (hippocampus), LH = lateral hypothalamus, PL = prelimbic cortex, IL = infralimbic cortex, NAcC = nucleus accumbens core, NAcS = nucleus accumbens shell, PVT = paraventricular nucleus of thalamus, STN = subthalamic nucleus, VP = ventral pallidum, VTA = ventral tegmental area. Generated using Biorender.

Across substance classes, we examine R/R under different extinction and abstinence conditions, including punishment-imposed abstinence, to highlight their clinical and translational relevance, citation incidence, and literature connectivity (circuits) of major terms in the field (Figures 2–4 and Table 1). Clinical studies are considered separately in the Discussion under a dedicated Clinical subsection, whereas non-human primate evidence is discussed in a separately labeled Non-Human Primates subsection. We then discuss mechanism-informed intervention strategies, evaluate pharmacological and behavioral approaches, identify key knowledge gaps, and propose future directions, including circuit-targeted approaches, improved integration of R/R paradigms, and the development of preclinical models more closely aligned with human relapse. Through this synthesis, we aim to clarify the roles of R/R in drug-associated relapse and to inform the development of more effective and durable interventions.

**Table 1:** Region involvement and drug coverage across renewal and reacquisition.

Region	Renewal	Reacquisition	Psychostimulant	Opioids	Alcohol	Nicotine	Cannabinoids
Basolateral amygdala	++	++	H	H	M	L	L
Dorsal hippocampus	+	+	M	M	M	L	M
Infralimbic cortex	+++, −	−	H	M	M	L	L
Insula	+	++	M	L	M	H	L
Lateral hypothalamus	+	++	M	M	H	M	L
Nucleus accumbens core	++	+++	H	M	M	M	L
Nucleus accumbens shell	+	++	M	H	M	L	M
Prelimbic cortex	++	+++	H	M	M	M	L
Ventral hippocampus	+++	+	H	M	M	L	M
Ventral pallidum	++	+++	H	M	M	M	L
Ventral tegmental area	++	+++	H	M	M	H	L

Symbols indicate change relative to the extinction state within the same subject. ‘+’ denotes participation, ‘++’ reliable contribution, and ‘+++’ necessary/causal involvement for relapse, whereas ‘−’ reflects potential (related) inhibitory control by extinction mechanisms; H/M/L indicate literature reproducibility for each drug class (high/moderate/limited).

## 2 Renewal and reacquisition (R/R): neurobehavioral insights

The expression and magnitude of R/R may vary across drug classes and depend on whether drug seeking is suppressed through extinction training or abstinence-based procedures, suggesting distinct forms or degrees of relapse shaped by neural circuitry, molecular adaptations, substance class, and behavioral history (Figure 4 and Table 1). As illustrated in Figure 3, separate bibliometric networks generated for reinstatement, renewal, and reacquisition highlight marked differences in the scope of the literature, with reacquisition represented by a substantially smaller and less interconnected body of work compared to reinstatement and renewal. Although fewer studies have directly examined reacquisition, particularly those manipulating specific neural circuits or molecular substrates, emerging work has begun to address behavioral determinants, including the influence of prior extinction history on subsequent reacquisition.

### 2.1 Behavioral modifiers of renewal and reacquisition

#### 2.1.1 Renewal behavioral modifiers

Renewing substance memories relies on contextual variants that are influenced by behavioral modifiers. This section covers several drug-specific scenarios in which these factors shape action output, although direct neurobiological evidence (circuit or molecular bases) remains limited.

During cocaine renewal, drug-paired contexts and cues (social or inanimate) can function similarly to conditioned stimuli following extinction. In support of this premise, both social and nonsocial contextual stimuli (e.g., light cues) can induce renewal of seeking; however, social cues make a predominant contribution, as omission of the social cue prevents renewal (Humburg and Bardo, 2025). This contrasts with other studies suggesting that social interactions oppose drug-seeking behaviors (El Rawas et al., 2020; Venniro et al., 2022). Moreover, renewal of cocaine seeking is modulated not only by cue type and context but also by developmental stage, as it is sensitive to age and incubation-like processes, with adolescents exhibiting time-dependent increases in cocaine renewal after prolonged abstinence (Charpentier et al., 2023; Olekanma et al., 2023).

Data on renewal following methamphetamine (METH) self-administration is sparse. Interestingly, Adhikary et al. (2017) reported that returning rats to the original drug-associated context produced robust renewal, although this context-induced effect did not incubate across withdrawal periods. However, the influence of biological factors such as age and sex on METH renewal and subsequent reacquisition remains largely unexplored.

As in METH literature, data on renewal following nicotine self-administration are limited. Notably, extinction effects on renewal appear to depend on a specific temporal window following reactivation of drug-associated memories. Extinction of nicotine-associated cues attenuates renewal only when extinction occurs within a narrow time window after cue reactivation, highlighting time-dependent sensitivity of nicotine-associated memories to extinction (Auber et al., 2014). Beyond temporal factors, socially relevant cues also modulate nicotine renewal. Some researchers demonstrated that pairing nicotine with olfactogustatory cues and carbon disulfide (CS₂), a rodent socially transmitted olfactory signal, enhanced nicotine seeking and renewal in social contexts, particularly at higher CS₂ doses (Wang and Chen, 2014). Together, these findings indicate that nicotine renewal is strongly influenced by extinction timing and social context, although comparatively fewer studies exist relative to other drugs of abuse.

Context plays a dominant role in the renewal of alcohol seeking (see Circuits and Molecular sections). ABA paradigms reliably elicit greater renewal than AAB or ABC designs (Khoo et al., 2020). Alcohol-associated cues increase responding mainly when presented in the original drug-paired context, underscoring the strong context dependence of cue–alcohol associations (Tsiang and Janak, 2006). When compared with natural rewards, alcohol seeking shows relatively greater persistence across repeated renewal tests, with responding remaining stable over extended periods following extinction, whereas sucrose seeking tends to diminish over time (Zironi et al., 2006). Notably, in rodents, extinction efficacy can be enhanced by pre-extinction memory reactivation: consumption of beer or non-alcoholic beer prior to extinction increases the latency of goal-seeking behavior (attenuated renewal) in an ABA design (Mason et al., 2024). This strategy has also produced positive effects on cocaine-seeking behavior, potentially through glutamatergic and dopaminergic mechanisms (Luo et al., 2015). Although the temporal constraints of this effect remain to be fully characterized, this finding is conceptually aligned with nicotine studies demonstrating time-dependent sensitivity of drug-associated memories to extinction.

Renewal of opioid seeking has been robustly demonstrated in heroin self-administration models using ABA. Re-exposure to heroin-paired contexts reliably reinstates drug-seeking behavior following extinction in a distinct environment, even in the absence of discrete cues (Crombag and Shaham, 2002). Together, these findings parallel renewal observed with cocaine and alcohol and underscore the dominant role of contextual memory in opioid relapse. Notably, whether similar renewal processes generalize to other opioids, including prescription or synthetic opioids, and across different biological variables or experimental designs remains largely unexplored.

Extending these observations to more complex substance histories, renewal has also been demonstrated following polydrug self-administration, although available evidence is limited. A study reported that rats trained to self-administer speedball (heroin–cocaine) exhibited a robust renewal selectively in an ABA context following extended extinction (Crombag and Shaham, 2002). However, systematic investigations of renewal across other polydrug combinations, patterns of use, abstinence conditions, and biological or experimental variables remain sparse.

Natural rewards can also engage R/R processes and, although the present review focuses on substance-related R/R, these studies underscore that renewal and reacquisition reflect fundamental learning and memory mechanisms rather than exclusively drug-specific phenomena. Examining natural rewards, therefore, provides an important comparison point for distinguishing general reinforcement-driven processes from adaptations more specific to drugs of abuse. For example, sucrose self-administration produces robust renewal in rats trained in one context and extinguished in another, paralleling findings observed with addictive substances (Pintori et al., 2022a; Todd et al., 2012). However, in contrast to cocaine self-administering rats, which show reduced responding following environmental enrichment (EE), sucrose self-administration rats exhibit increased discrimination for the active lever during context-induced sucrose-seeking tests (Ranaldi et al., 2011; Pintori et al., 2022a). However, an interesting dynamic was found in mice where brief EE decreased test responding to the levels of extinction compared to non-EE groups, who significantly increased their responding. An effect seen in both socially isolated and socially housed mice (Pintori et al., 2022b). Comparing the dissimilar effects in rodent models of sucrose renewal indicates a different mechanism (i.e., circuit and molecular processes) between natural and drug rewards, as both rats and mice exhibit robust renewal post-extinction with drugs of abuse.

#### 2.1.2 Reacquisition behavioral modifiers

As reacquisition relies less on contextual variants and more on drug availability, this section covers several drug-specific scenarios in which behavioral modifiers shape action output and reacquisition sequences with limited neurobiological evidence.

It has been shown that extinction history exerts minimal influence on overall reacquisition magnitude (Willcocks and McNally, 2011). Rats with and without extinction histories showed comparable total responding during reacquisition (alcohol), although extinction transiently reduced early-session responding. Extending extinction duration produced only a non-significant increase in response latency, and extinction did not prevent reacquisition across contexts. While reacquisition occurred regardless of context, response initiation remained context sensitive, with faster responding in the original drug context. Motivational testing using progressive ratio schedules revealed that extinction history reduced breakpoints relative to non-extinguished rats; however, all reacquiring animals exhibited higher breakpoints than those during initial acquisition, indicating that reacquisition reflects a more efficient re-engagement of alcohol seeking once reinforcement is restored (Willcocks and McNally, 2011). A subsequent study also demonstrated that extinction retrieval cues attenuate renewal but do not suppress reacquisition of alcohol seeking once alcohol availability is restored. This dissociation persisted regardless of whether the extinction cue was contingent on instrumental responding (Willcocks and McNally, 2014). Together, these findings underscore the need to develop and refine behavioral strategies that more effectively promote transfer and generalization of extinction learning, particularly to prevent relapse driven by reacquisition, which appears largely insensitive (after the initial delay) to extinction history once drug availability is restored.

Beyond alcohol, reacquisition has been reported for other substances of abuse (see next sections), including cocaine and opioids, where re-exposure to the drug rapidly restores instrumental responding following extinction (e.g., Grimm et al., 2001; Bossert et al., 2020). However, most studies examining reacquisition of cocaine, opioids, nicotine, or cannabinoids have not systematically manipulated contextual variables, limiting direct comparison with R/R paradigms. As a result, the extent to which reacquisition across drug classes is modulated by context remains an important gap in the literature.

### 2.2 Neural circuits underlying R/R

Relapse driven by R/R emerges from partially overlapping but dissociable neural circuits that differentially engage contextual memory retrieval versus reinforcement re-engagement (Khoo et al., 2017). While many relapse modalities converge on mesolimbic structures, renewal shows heightened sensitivity to contextual information processing and memory networks, whereas reacquisition more strongly recruits motivational and action–outcome circuitry.

#### 2.2.1 Circuits supporting renewal

A core node in renewal is the nucleus accumbens shell (NAcS). Pharmacological inactivation of the NAcS selectively attenuates context-induced reinstatement of cocaine, heroin, and alcohol seeking without affecting cue- or drug-primed reinstatement, whereas the nucleus accumbens core (NAcC) is broadly required across relapse modalities (Fuchs et al., 2008; Chaudhri et al., 2010; Bianchi et al., 2024). Following extinction in a neutral context, re-exposure to the cocaine-associated context preferentially induces c-Fos expression in a sparse, predominantly medium spiny neuronal ensemble within the NAcS. Selective ablation of these extinction- and context-activated Fos-expressing neurons disrupts renewal, demonstrating the involvement of a specific neuronal ensemble in this process and its contribution to the retrieval and behavioral expression of context-drug associations (Cruz et al., 2014). These findings further suggest that the role of these ensembles extends beyond simple contextual dependence (e.g., via generalization processes), as renewal occurs in the drug-associated context rather than the extinction context, and highlight a potential avenue for future extinction interventions aimed at reducing renewal and limiting relapse vulnerability.

The hippocampus is a major upstream driver of renewal, consistent with its role in contextual memory processing. Inactivation of dorsal hippocampal (dHipp) subregions blocks renewal without affecting cue-induced reinstatement, while ventral hippocampal (vHipp) and ventral subiculum (vSub) manipulations similarly attenuate context-induced cocaine (Fuchs et al., 2005; Lasseter et al., 2010) and heroin (Bossert and Stern, 2014) seeking. Chemogenetic dissection further demonstrates that vSub → NAcS projections are necessary for renewal of alcohol and heroin seeking, positioning this pathway as a principal conduit for contextual information into accumbal circuits (Marchant et al., 2016). Cholinergic modulation of this pathway via M4 muscarinic receptors further regulates renewal magnitude, highlighting neuromodulatory control over contextual relapse (see the molecular section) (Walker et al., 2021).

Renewal also depends on coordinated interactions between the dHipp, basolateral amygdala (BLA), and the medial prefrontal cortex (mPFC). Pharmacological inactivation or contralateral disconnection of the dHipp, BLA, and PL disrupts renewal of cocaine seeking, indicating that interregional communication is required (Fuchs et al., 2005, 2007). However, drug-specific differences emerge within the mPFC. In heroin models, infralimbic (IL; ventral mPFC) activity and its projections to the NAcS are critical for renewal, whereas prelimbic (PL; dorsal mPFC) involvement appears more prominent in alcohol and cocaine renewal (Bossert et al., 2011, 2012; Fuchs et al., 2005; Bianchi et al., 2024; Tavares et al., 2023). These findings suggest that prefrontal control over renewal varies by drug class and projection target.

The BLA integrates contextual, affective, and neuromodulatory signals to support renewal. Local blockade of *μ*-opioid or glucocorticoid receptors within the BLA attenuates renewal of alcohol and cocaine seeking, suggesting that stress- and opioid-related signaling within the BLA contributes to the retrieval and behavioral expression of context–drug associative memories. Notably, glucocorticoid receptors are activated by stress-induced release of glucocorticoids (e.g., corticosterone), which modulate neuronal excitability and synaptic plasticity within the BLA, thereby facilitating the retrieval of emotionally salient, context-drug memories (Marinelli et al., 2009; Yuan et al., 2020). More recently, corticotropin-releasing factor (CRF) signaling has been identified as a critical molecular input to this circuit: inhibition of dorsal raphe CRF neurons projecting to the BLA during memory reactivation weakens subsequent context-induced cocaine seeking, implicating a CRF-dependent reconsolidation mechanism. Such a system may be the process by which a reactivated memory becomes temporarily labile and must be restabilized to persist within the renewal circuitry (Ritchie et al., 2024) (see also the molecular section).

Additional regions modulate renewal by shaping motivational state or contextual salience. The paraventricular thalamus (PVT) exhibits increased activity during renewal, and lesions reduce alcohol seeking, potentially via dopaminergic signaling within the NAcS (Hamlin et al., 2009; Parsons et al., 2007). Inactivation of cortical regions, including the lateral orbitofrontal cortex (lOFC) and insular cortex contribute to attenuating renewal of cocaine and nicotine seeking (Lasseter et al., 2009; Arguello et al., 2017; Ghareh et al., 2022). Conversely, the dorsolateral septum appears to exert inhibitory control over renewal, as its inactivation enhances cocaine seeking, revealing a potential brake on reconsolidation processes (Qi et al., 2025).

Downstream of the accumbens, the ventral pallidum (VP) serves as a major output structure. Although NAcC → VP projections are recruited during renewal, functional manipulations indicate that VP pathways to the lateral hypothalamus (LH) rather than to the ventral tegmental area (VTA) are selectively required for alcohol renewal expression (Perry and McNally, 2013a; Prasad et al., 2020). The LH integrates inputs from the NAc and VP to balance opposing motivational signals, positioning it as a critical node for contextual relapse expression (Gibson et al., 2018).

#### 2.2.2 Circuits supporting reacquisition

In contrast to renewal, reacquisition relies more heavily on reinforcement-driven circuits that rapidly re-engage drug-taking behavior once the drug becomes available. Most evidence comes from alcohol studies, which, as illustrated in Figure 2, represent a disproportionate share of the R/R literature, potentially limiting generalization to other substances, but consistently implicates signaling within the NAcC-VP axis. Fiber photometry and optogenetic studies demonstrate that both medial and lateral VTA dopamine neurons contribute to reacquisition, with renewal associated with greater recruitment of lateral VTA populations (Liu et al., 2020). Correspondingly, reacquisition elicits robust dopamine transients in accumbal subregions resembling initial acquisition rather than extinction or renewal states (Liu et al., 2020) (see Figure 4 for a broader circuit schematic).

Cell-type-specific circuits within the VP distinctly dissociate reacquisition from renewal. Parvalbumin-expressing VP neurons projecting to the VTA are required for both renewal and reacquisition, whereas VP GABAergic projections to the LH selectively regulate renewal (Prasad et al., 2020). These findings indicate that reacquisition depends on VP–VTA circuits that amplify motivational drive and reinforcement learning rather than contextual memory retrieval. Also, different from renewal, inhibition of the NAcC → VP pathway reduces reacquisition of alcohol (Khoo et al., 2015), further suggesting divergent roles supporting renewal and reacquisition.

Prefrontal contributions to reacquisition are comparatively modest. In alcohol models, PL inhibition paradoxically augments reacquisition while attenuating renewal, suggesting that extinction-related cortical control may constrain drug taking only in the absence of reinforcement (Willcocks and McNally, 2013). Together, these data indicate that reacquisition emerges from subcortical reinforcement circuits that can bypass cortical inhibitory control once the substance is available.

### 2.3 Molecular mechanisms underlying R/R

The molecular processes that shape the expression and magnitude of R/R may differ across substances and depend on whether drug seeking is suppressed through extinction or abstinence. While some molecular mechanisms of renewal have been identified, this work remains limited, and the molecular basis of reacquisition has received even less attention (Figure 3). More studies are needed to clarify how shared and distinct molecular (i.e., transcriptomic and epigenetic) pathways contribute to R/R across different substances and behavioral histories.

#### 2.3.1 Molecular mechanisms supporting renewal

At the molecular level, renewal is supported by mechanisms that stabilize and retrieve drug–context memories rather than re-initiate reinforcement learning. Collectively, these findings align with reconsolidation frameworks in which brief reactivation of drug-associated memories renders them labile and susceptible to molecular updating during extinction or abstinence, rather than erasure (Tronson and Taylor, 2007).

Dopamine signaling, particularly via D1-family receptors, plays a central role in renewal, with evidence for subregion-specific effects. Blockade of D1 receptors in the NAcS attenuates renewal of heroin and alcohol seeking, whereas NAcC D1 signaling preferentially supports cue-driven relapse (Bossert et al., 2007; Chaudhri et al., 2009; Hamlin et al., 2007). Renewal-associated Fos induction in the NAcS and LH further supports a preferential engagement of shell-directed dopamine signaling (Hamlin et al., 2007; Perry and McNally, 2013a).

Endogenous opioid systems modulate renewal by regulating affective and cue components of relapse. *μ*- and *δ*-opioid receptor antagonism reduces context-induced alcohol seeking across multiple limbic regions, including the BLA, NAcS, and VP, with distinct temporal contributions from each receptor class (Marinelli et al., 2007b, 2009; Perry and McNally, 2013b). These effects are consistent with opioid modulation of memory retrieval and motivational valuation of drug-associated contexts.

Stress-related neuromodulators critically regulate renewal through their effects on memory reconsolidation. CRF signaling within the BLA is required for cocaine-memory maintenance, and selective inhibition of dorsal raphe CRF projections to the BLA during memory reactivation weakens subsequent renewal (see also the Circuits section; Ritchie et al., 2021, 2024). Together with evidence implicating glucocorticoid signaling in renewal, these findings position stress peptides as central regulators of context-driven relapse.

Glutamatergic plasticity within hippocampal and amygdalar circuits further supports renewal. Inhibition of NMDA receptor signaling, Src-family kinases, ERK signaling, or protein synthesis within the dHipp or BLA selectively disrupts reconsolidation-dependent renewal of cocaine seeking without affecting extinction performance (Wells et al., 2013, 2016; Arguello et al., 2014; Xie et al., 2012). These molecular cascades appear necessary for maintaining contextual drug memories following reactivation.

#### 2.3.2 Molecular mechanisms supporting reacquisition

Reacquisition is supported by molecular signaling that enables rapid re-engagement of reinforcement and motivation once the substance becomes available. Dopamine-dependent processes within mesolimbic pathways restore accumbal dopamine dynamics resembling initial drug taking, while downstream regions, including the VP and LH, preferentially amplify reinforcement-related output during reacquisition relative to renewal. Circuit and cell/circuit-type specificity are detailed in the Circuits section (Liu et al., 2020; Prasad et al., 2020). However, the molecular substrates distinguishing reacquisition from renewal remain incompletely characterized.

Opioid-based pharmacotherapies exert variable effects on reacquisition that depend on substance class, sex, and receptor bias. Buprenorphine analogs, nociceptin receptor (NOR) ligands, and μ-opioid receptor (MOR)–biased agonists selectively modulate reacquisition of heroin and oxycodone, often producing sex-dependent outcomes (Bossert et al., 2020, 2022, 2024). It’s found that the mixed NOP/MOR partial agonist AT-201 increased heroin reacquisition in females, whereas the NOP antagonist J-113397 reduced the incubation of heroin seeking (rather than reacquisition) (Bossert et al., 2024). In related work, the same group showed that the buprenorphine analogue BU08028 also increased heroin reacquisition in female, but not male, rats (Bossert et al., 2022). Lastly, they reported that buprenorphine decreased reacquisition of oxycodone self-administration in both sexes, whereas TRV130 (a MOR agonist) produced this effect only in males (Bossert et al., 2020). These results suggest that reacquisition engages opioid systems primarily through reinforcement valuation rather than memory mechanisms.

Across stimulant models, reacquisition is highly sensitive to interventions that enhance extinction-related plasticity. EE, glutamatergic modulation, and activity-dependent remodeling within NAc–mPFC circuitry consistently attenuate cocaine reacquisition, often more robustly than renewal (Chauvet et al., 2012; Gauthier et al., 2017; Hastings et al., 2020; Kantak et al., 2020; Peterson et al., 2024). EE prevents the development of cocaine craving and reverses incubation, but these effects are transient and dissipate when enrichment is discontinued, indicating that sustained exposure is required during periods of relapse vulnerability (Chauvet et al., 2012). Mechanistically, EE enhances extinction efficacy through coordinated, temporally specific modulation of AMPAR GluA1 phosphorylation within vmPFC–amygdala circuits and TrkB-dependent plasticity in vmPFC, NAc, BLA, and dHipp that oppose reacquisition, changes not observed with extinction or enrichment alone (Gauthier et al., 2017; Hastings et al., 2020; Peterson et al., 2024). In addiction-like phenotypes induced by high cocaine doses, combined EE and GlyT-1 inhibition suppresses reacquisition in males but not females, despite facilitating extinction in females, revealing a sex-specific dissociation between extinction enhancement and relapse prevention that may reflect reduced excitatory signaling and TrkB expression in females (Kantak et al., 2020; Peterson et al., 2024).

Additional molecular mechanisms implicated in R/R include dopamine, opioid, CRF-related signaling, and synaptic plasticity pathways, where these mechanisms vary by drug class and experimental conditions and can be modulated by sex (Becker and Koob, 2016). In addition, punishment-based abstinence models reveal partially distinct molecular signatures compared with extinction-based paradigms, underscoring the need for systematic comparisons across relapse forms, sexes, and abstinence conditions.

### 2.4 R/R and punishment-imposed abstinence

Extinction serves as the underlying mechanism for abstinence in many preclinical models, involving both the updating of response-outcome contingencies and the formation of context-dependent inhibitory memories following reward omission. In contrast, punishment-imposed abstinence suppresses drug seeking by establishing a competing response-outcome association in which the action produces an aversive consequence, thereby engaging partially distinct motivational and neural processes. Work in alcohol-preferring rats demonstrated that alcohol-associated contexts can robustly reinstate seeking even when abstinence is enforced by adverse consequences rather than extinction, highlighting the dominant role of contextual cues over relapse behavior (Vengeliene et al., 2009; Marchant et al., 2013). For instance, it’s reported that punishment suppressed alcohol taking only when shocks were response-contingent, and relapse occurred selectively upon re-exposure to the alcohol-paired context, indicating that punishment learning is context-specific rather than globally suppressive of wanting behaviors (Marchant et al., 2013).

Similarly, punishment-based models (not ABA) reveal substantial individual differences in the ability to suppress substance use in the face of adverse consequences, providing insight into compulsive drug taking (Hu et al., 2019). In a methamphetamine self-administration model, rats escalated intake during acquisition and reduced responding when lever presses were paired with response-contingent foot shock, yet punishment uncovered distinct shock-resistant and shock-sensitive subgroups (Hu et al., 2019). This is also consistent with other studies that link this resistance to mPFC hypofunction (Chen et al., 2013; Pelloux et al., 2007). These groups did not differ in total methamphetamine intake or seeking behavior prior to punishment, indicating that vulnerability to compulsive-like drug taking could not be predicted from pre-punishment drug exposure alone. Instead, compulsivity emerged selectively during punishment, with shock-resistant rats maintaining higher levels of drug taking despite escalating adverse consequences, a behavioral phenotype correlated with strengthened orbitofrontal cortex-dorsomedial striatal “go” circuit engagement and altered balance with prefrontal “stop” circuitry.

Building on this framework, Pelloux and colleagues extended punishment-induced abstinence paradigms to cocaine self-administration (Pelloux et al., 2018a). Rats trained to self-administer cocaine in one context subsequently underwent punishment-imposed abstinence in a distinct context, where response-contingent footshock suppressed cocaine seeking. When returned to the cocaine-paired context, robust renewal was observed in animals that experienced contingent punishment, demonstrating context specificity. cFos mapping revealed a neural profile unique from that observed in alcohol models. Whereas renewal of punished alcohol seeking depends on dopamine D1-family receptor signaling within the NAc (Marchant et al., 2014; Vengeliene et al., 2009), cocaine renewal following punishment was not associated with NAc activation (although a non-significant increase in NAcC activity was observed). This cocaine renewal with punishment history recruited a broader network, including mPFC, agranular insula cortex (aIC), dorsal striatum, BLA, PVT, vSub, and dorsal raphe (Pelloux et al., 2018b). These findings indicate that punishment-resistant relapse engages circuit mechanisms that differ fundamentally from extinction-based renewal and vary across drug classes.

This dissociation aligns with evidence that compulsive substance use is associated with an imbalance between prefrontal, striatal, amygdalar, and other reward and affective-related regions. Pelloux et al., demonstrated that individuals who persist in drug seeking despite punishment exhibit disrupted prefrontal control over striatal output, providing a mechanistic structure for punishment-resistant renewal (2018b). They found that temporary inactivation of the BLA or CeA attenuated context-induced cocaine seeking following extinction, whereas under punishment conditions, BLA inactivation selectively enhanced renewal irrespective of the context history and inactivation of either region elicited renewal in the punished context, indicating that amygdalar contributions to context-induced relapse vary as a function of the abstinence procedure (Pelloux et al., 2018b). Consistent with this interpretation, comparative analyses of extinction- versus punishment-induced abstinence confirm that these abstinence mechanisms recruit overlapping but partially dissociable neural substrates (Marchant et al., 2019), underscoring the importance of studying punishment paradigms as a complementary model of relapse vulnerability. However, whether punishment during renewal testing recruits distinct brain regions, or whether prior extinction history alters the impact of punishment during renewal, reacquisition, or approach/avoidance sessions and decisions through the engagement of different neural circuits, remains to be determined.

Additional studies in alcohol-preferring rats reveal that punishment history can shape relapse vulnerability across biological dimensions. Male offspring of alcohol-experienced sires displayed attenuated context-induced relapse following punishment-imposed abstinence, despite preserved renewal effects, suggesting intergenerational and genetic modulation of punishment sensitivity or contextual valuation of alcohol-associated cues (Campbell et al., 2018). Complementary work examining sex differences showed that females were less likely than males to resume alcohol seeking in a punishment-associated context after prolonged abstinence, even though both sexes exhibited comparable alcohol intake during training (Campbell E. J. et al., 2021). These findings further highlight the influence of sex and individual differences in punishment-based relapse models.

Dopamine signaling remains a critical modulator of relapse after punishment for alcohol, but not uniformly across drugs. Pharmacological blockade of D1-family receptors in the NAcC or NAcS attenuates renewal following punishment of alcohol seeking, reinforcing the central role of accumbal dopamine in alcohol relapse (Marchant and Kaganovsky, 2015). In contrast, cocaine relapse following punishment is less accumbens dependent, consistent with a shift toward cortical and associative control systems under conditions of aversive conflict (Pelloux et al., 2018a).

Punishment-based relapse mechanisms have also been examined in nicotine models. Activity in the aIC is necessary for context-induced relapse following punished nicotine seeking, with relapse-associated activation observed in aIC neurons and BLA inputs to the aIC (Ghareh et al., 2022). Chemogenetic inhibition of the aIC reduced nicotine relapse after punishment as well as after extinction, suggesting that insular cortex function generalizes across abstinence mechanisms while still supporting context-driven relapse.

Collectively, these studies demonstrate that renewal following punishment-imposed abstinence and its associated relapse and reacquisition processes are mechanistically distinct, at least in part, from extinction-based R/R. Punishment-based relapse recruits partially overlapping but drug-specific circuits, is strongly shaped by response–outcome contingencies, and reveals dimensions of relapse vulnerability related to compulsion, sex, and individual differences. Notably, whereas accumbens dopamine signaling plays a central role in punished alcohol relapse, relapse to cocaine and nicotine after punishment preferentially engages distributed cortical–subcortical (including dorsal striatum) networks implicated in action selection, interoception, and aversion processing (Pelloux et al., 2018a, 2018b; Marchant et al., 2019; Jonkman et al., 2012). Together, these findings highlight critical gaps in understanding how punishment history interacts with contextual control, circuit engagement, biological variables, and drug class to govern relapse vulnerability, and whether prior extinction training or forced abstinence modifies renewal and/or reacquisition when relapse testing occurs under punishment conditions.

## 3 Discussion

### 3.1 Clinical and translational relevance

Relapse in SUDs remains a major clinical challenge, in part because abstinence-oriented treatments often fail to address the strong context dependence of drug-seeking behavior. Preclinical R/R models demonstrate that extinction or punishment does not erase drug-associated memories but instead establishes inhibitory learning that is fragile and highly context specific. This framework closely mirrors clinical relapse, which frequently occurs when individuals return to drug-associated environments following treatment, incarceration, or hospitalization. Within this translational lens, renewal models capture relapse driven by contextual re-exposure in the absence of substance availability, whereas reacquisition models reflect the rapid resumption and escalation of drug use once access is restored.

#### 3.1.1 Context dependence and generalization of treatment effects

Exposure-based treatments, including cue-exposure therapy, have shown limited long-term efficacy in clinical populations, likely due to poor generalization of extinction learning across contexts. Human neuroimaging studies consistently demonstrate that drug-associated cues and environments engage hippocampal–striatal–prefrontal networks implicated in renewal in animal models, and that the magnitude of activation within these circuits predicts craving intensity and relapse risk (Kosten et al., 2006; Chase et al., 2011; Sinha et al., 2005; Volkow et al., 2003, 2007; Volkow and Baler, 2013). These findings parallel extensive preclinical evidence stressing the need for treatments that promote context generalization (or transfer) rather than context-specific extinction. Clinical observations further support this view: relapse risk is disproportionately elevated when individuals return to environments previously associated with substance use, even after prolonged abstinence (Crombag et al., 2008). Together, these data suggest that relapse vulnerability reflects persistent context–memory associations, aligning closely with renewal mechanisms described in animal models.

#### 3.1.2 Punishment, abstinence, and real-world relapse

Punishment-imposed abstinence models provide a relevant translational view, as many individuals reduce or discontinue drug use due to adverse consequences rather than extinction-based learning. Clinical evidence indicates that legal, social, and health-related consequences often fail to prevent relapse once individuals return to drug-associated contexts, consistent with preclinical findings showing robust context-induced relapse after punishment (Hu et al., 2019; Marchant et al., 2013).

Human studies of compulsive substance use further suggest that punishment-resistant individuals exhibit dysregulated prefrontal–striatal control and impaired evaluation of adverse outcomes (Ersche et al., 2016; Myers et al., 2017; Enzi et al., 2015; Luijten et al., 2013). These findings converge with animal studies demonstrating that relapse after punishment recruits circuits involved in action selection, interoception, and aversion processing rather than classic reward circuitry alone (Hu et al., 2019; Chen et al., 2013; Pelloux et al., 2007). Importantly, these observations support the idea that relapse vulnerability reflects deficits in cognitive and circuit-level control mechanisms, not simply heightened reward sensitivity; however, our understanding of how punishment history shapes these control processes during subsequent relapse remains limited.

Extending these findings (not ABA), related translational opioid work using consequence-driven abstinence procedures has shown that oxycodone seeking can be potentiated following abstinence induced by adverse outcomes. Imaging studies demonstrate that oxycodone self-administration in rodents increases orbitofrontal cortex–dorsal striatal functional connectivity, whereas electric barrier–induced abstinence decreases connectivity within these circuits; importantly, although this procedure involves an element of choice, abstinence is effectively imposed by progressively increasing shock intensities (Fredriksson et al., 2021). Both phases predict subsequent incubation of oxycodone craving. In parallel, pharmacological evidence shows that the dopamine stabilizer (−)-OSU6162 reduces incubated oxycodone seeking following electric barrier–induced abstinence, supporting a role for dopamine-related mechanisms in this form of relapse vulnerability (Fredriksson et al., 2020). However, how these circuit and neurochemical adaptations interact with punishment history to shape control processes during relapse remains unclear.

#### 3.1.3 Circuit-informed targets for intervention

Circuit-level dissociations between R/R suggest that distinct therapeutic strategies may be required to prevent relapse versus limit escalation once relapse occurs. Context-driven (memory) relapse preferentially engages hippocampal and amygdalar inputs to the NAcS, with strong modulation by stress-related signaling and dopaminergic tone. In contrast, reacquisition more robustly recruits core mesolimbic dopamine output pathways, particularly VP and VTA circuitry that drives reinforcement and rapid re-escalation of drug seeking.

Human imaging studies support this distinction, showing that striatal dopamine-related activity predicts relapse severity and speed of return to drug use (Versace et al., 2014; Courtney et al., 2016). Together, these findings argue for mechanism-informed interventions in which targeting contextual memory retrieval or reconsolidation may reduce relapse vulnerability, while dampening dopaminergic drive or downstream motivational circuits may limit rapid re-escalation of use once relapse occurs.

#### 3.1.4 Clinical evidence supporting context- and memory-based interventions

Consistent with preclinical findings, human studies provide emerging evidence that manipulating memory retrieval and extinction processes can reduce craving and drug use (not necessarily with the ABA design). In smokers, a retrieval–extinction (R–E) procedure significantly reduced cigarette craving and consumption (Germeroth et al., 2017). Following brief abstinence and cue-induced craving assessment, participants in the R-E group viewed a smoking-related memory retrieval video prior to extinction training across two consecutive days, whereas the no retrieval-extinction (NE-R) group viewed a neutral video before undergoing the same extinction procedures. At follow-up (24 h, 2 weeks, and 1 month), the R-E group reported smoking fewer cigarettes per day, showed a greater proportion achieving substantial reductions in smoking, and exhibited lower expired carbon monoxide levels at 1 month. Craving effects were modest overall, but the R-E group demonstrated reduced craving at the final test session, suggesting enhanced durability of extinction when paired with memory retrieval.

Parallel findings have been reported in other substance-using populations. In heroin-dependent individuals, retrieval–extinction procedures inspired by the Monfils effect (Monfils et al., 2009) reduced cue-induced craving and relapse-related responding by targeting substance memories during a labile reconsolidation window, providing early clinical support for reconsolidation-based interventions (Xue et al., 2012). However, subsequent work indicates that the effectiveness of retrieval–extinction paradigms is not uniform across drugs or behavioral contexts, with evidence that nicotine seeking but not cocaine seeking can be resistant to this manipulation in operant self-administration models (Struik et al., 2019). Complementary studies have begun to elucidate the neural mechanisms underlying retrieval–extinction and reconsolidation-based effects. For example, post-retrieval extinction in methamphetamine-trained rats reduced context- and cue-induced relapse as well as spontaneous recovery and was associated with altered activation of prefrontal–amygdala circuits implicated in relapse control (Chen et al., 2019). In addition, causal circuit-level evidence demonstrates that optogenetic inhibition of dorsal hippocampal CA3 neurons during early-stage cocaine-memory reconsolidation selectively disrupts subsequent context-induced cocaine seeking, highlighting a critical role for hippocampal memory processes in relapse vulnerability (Qi et al., 2022). Consistent with this idea, early extinction-based cue-exposure studies in cocaine-dependent individuals demonstrated reductions in subjective craving and improved treatment retention despite persistent physiological cue reactivity (O’Brien et al., 1990). Together, these findings suggest that weakening cue–drug associations during periods of memory lability can reduce relapse vulnerability without fully erasing the original drug memory, while also underscoring the importance of drug class, behavioral context, and circuit engagement in determining the effectiveness of reconsolidation- and extinction-based interventions.

#### 3.1.5 Bridging evidence from non-human primates

Although formal ABA-style R/R paradigms have not yet been systematically implemented in non-human primates (NHPs), this absence reflects a translational gap rather than a lack of relevance. NHP studies robustly demonstrate that drug-associated contexts and discriminative stimuli exert strong control over drug seeking, and that drug taking can persist despite escalating response costs or adverse consequences, indicating punishment resistance and individual variability in behavioral control (Nic Dhonnchadha et al., 2010; Johanson, 1978; Woolverton and Johanson, 1984; Negus, 2005; Weerts et al., 2006). At the circuit level, NHP research consistently implicates prefrontal–striatal and orbitofrontal networks in regulating drug seeking, decision-making, and sensitivity to negative outcomes, networks that closely parallel those identified in rodent punishment models and human neuroimaging studies (Lucantonio et al., 2012; Goldstein and Volkow, 2011; Marchant et al., 2019; Porrino et al., 2016; Porrino et al., 2023; Broomer and Bouton, 2023). In line with this work, a longitudinal NHP study indicated that prolonged abstinence could increase sensitivity to cocaine reinforcement in adulthood, particularly following early life stress (Allen et al., 2025). These effects likely reflect stress-dependent alterations in prefrontal–striatal regulation of drug-seeking behavior that converge with mechanisms identified in rodent punishment and incubation models. Complementing these behavioral and circuit-level findings, transcriptomic analyses in rhesus macaques following prolonged cocaine self-administration reveal region-specific molecular adaptations within the mesolimbic system, including coordinated down-regulation of dopaminergic gene programs in the VTA and robust immune- and epigenetic-associated transcriptional remodeling in the NAc (Vallender et al., 2017). These molecular signatures closely parallel those observed in human post-mortem tissue (Bannon et al., 2005; Mews et al., 2023) and support the idea that chronic substance exposure produces enduring alterations in prefrontal–striatal and mesolimbic circuits that may bias behavior toward persistent, punishment-resistant drug seeking. Together, these cross-species findings position rodents as mechanistically precise models for dissecting R/R circuitry, NHPs as essential intermediates for validating circuit homology and behavioral control mechanisms, and humans as the clinically expressive endpoint in which relapse unfolds within complex cognitive, emotional, and social contexts.

### 3.2 Toward mechanism-informed interventions and future directions

A central implication of R/R research is that relapse vulnerability arises from identifiable behavioral, circuit, and molecular mechanisms that are insufficiently targeted by current treatments. Extinction- and punishment-based interventions suppress drug seeking without erasing underlying drug-associated memories, leaving relapse highly sensitive to context, stress, and renewed drug availability (Bouton, 2002; Kalivas and O’Brien, 2008). Mechanism-informed interventions should therefore aim to (i) reduce the contextual specificity of inhibitory learning, (ii) weaken or update maladaptive drug memories, and (iii) restrict rapid re-engagement of substance use once relapse is initiated.

#### 3.2.1 Behavioral strategies

Behavioral interventions that enhance generalization or transference of extinction or punishment learning across contexts represent a critical translational priority. Preclinical work demonstrates that extinction learning is context-bound, whereas R/R reflect preserved excitatory drug memories that dominate when inhibitory control fails (Bouton, 2002; Crombag et al., 2008). Approaches that vary extinction contexts, pair extinction with memory retrieval, or strategically incorporate punishment contingencies or alternative rewards can reduce context specificity and attenuate relapse vulnerability (Todd et al., 2014; Pelloux et al., 2018a).

Social variables also represent a particularly powerful but underutilized behavioral dimension. Rodent studies show that social interaction can compete with drug-associated contextual memories and robustly suppress relapse, even after prolonged abstinence (El Rawas and Saria, 2016; Salti et al., 2015; Venniro et al., 2021, 2022, 2018; Marcus et al., 2022). These protective effects are particularly evident in paradigms such as dyadic social interaction, social choice, and social enrichment, especially when the social partner has no prior drug exposure, highlighting the importance of social context in modulating drug-related behavior (Smith, 2012). Convergent human evidence demonstrates that social reinforcement and structured social support reliably reduce relapse risk across substances, including cocaine, opioids, alcohol, and nicotine. Interventions such as contingency management, community reinforcement approaches, and peer-based recovery programs improve abstinence duration and treatment retention (Higgins et al., 2002, 2004; Moos and Moos, 2006; Kelly et al., 2017). Neuroimaging studies further show that social reward and social stress recruit limbic–striatal–prefrontal circuits overlapping with drug-cue–responsive networks, including the ventral striatum (NAc), aIC, and mPFC (Eisenberger, 2012; Sinha et al., 2004, 2005). Along with social support, EE also appears to be a strong modulator of drug-seeking behaviors. For instance, EE attenuated the reinstatement of cocaine seeking induced by cues and stress, but not by cocaine priming, following extinction (Chauvet et al., 2009). Consistent with this, EE prior to extinction similarly reduced renewal, with EE rats displaying a steeper decline in active lever pressing during extinction and reduced renewed responding (Ranaldi et al., 2011). Collectively, these findings suggest that social and enriched reinforcement can function as competing motivational systems that attenuate context-driven relapse and strengthen extinction memory (when combined) yet remain insufficiently incorporated into formal relapse models.

#### 3.2.2 Emerging and experimental interventions

Pharmacological strategies that target stress systems, dopaminergic signaling, and memory reconsolidation offer promising adjuncts to behavioral treatment. For instance, dextroamphetamine has emerged as a promising agonist-replacement approach that reduces cocaine intake across preclinical, NHP, human laboratory, and clinical contexts (Peltier et al., 1996; Negus and Mello, 2002; Chiodo et al., 2008; Czoty et al., 2011), and recent rodent work further indicates that dextroamphetamine can limit cocaine seeking during reacquisition (Smith et al., 2024).

Preclinical studies also demonstrate that CRF, glucocorticoid, and kappa/dynorphin signaling within relapse circuits modulate (i.e., enhance) drug-associated memories during context re-exposure or reinstatement procedures (Marinelli et al., 2007a; Ritchie et al., 2024; Logrip and Gainey, 2020; Wang et al., 2008; Bloodgood et al., 2021; Chavkin and Koob, 2016; Mantsch et al., 2016). In humans, stress hormones strongly predict cue- and context-induced craving, relapse risk, and treatment failure across substances (Sinha et al., 2005; Preston et al., 2018; Marchette et al., 2025), supporting translational interest in stress-based interventions to modulate habit-like behaviors and decisional flexibility (i.e., approach/avoidance biases) (Sequeira et al., 2024).

In parallel, emerging evidence suggests that psychedelic compounds may facilitate relapse-relevant learning processes by enhancing synaptic plasticity, cognitive flexibility, and large-scale network reorganization. Human neuroimaging and clinical studies indicate that psychedelic-assisted therapies can reduce substance use and craving, potentially by weakening rigid maladaptive memory networks and increasing sensitivity to environmental context and therapeutic learning (Bogenschutz et al., 2015; Ly et al., 2018; Zafar et al., 2023). From a R/R perspective and its applicability in animals, these agents may be particularly effective when paired with structured behavioral interventions, rather than as stand-alone treatments, to promote durable updating of drug-associated memories. Notably, although rodent studies have expanded rapidly (Odland et al., 2022), the limited application of these approaches in addiction models represents a major barrier to translational progress.

Invasive interventions can also aim to attenuate R/R processes and the subsequent relapse. Childs et al. showed that vagus nerve stimulation (VNS) during extinction facilitated learning and reduced renewal, whereas VNS in the absence of lever availability had no effect (Childs et al., 2019). By strengthening extinction memory and its contextual control, VNS appears to primarily target renewal mechanisms, with less evidence that it limits reacquisition. Complementary studies further support extinction-augmenting interventions as effective in reducing relapse driven by cues or stress (Bremner et al., 2023; Alvarez-Dieppa et al., 2025; Shivaswamy et al., 2022). Deep brain stimulation (DBS) represents another invasive intervention for treatment-resistant SUDs, with demonstrated efficacy in clinical and translational studies (Yuen et al., 2022). Although no studies have directly examined DBS effects on renewal or reacquisition, existing evidence suggests that DBS reduces relapse-like behavior (e.g., reinstatement) and may enhance extinction-related processes that oppose relapse vulnerability (Luigjes et al., 2012; Martínez-Rivera et al., 2016; Lloret-Torres and Barreto-Estrada, 2025).

#### 3.2.3 Emerging molecular and cellular targets

Advances in circuit-specific genetic and molecular tools have revealed that R/R depend on pathways embedded within defined neural circuits, including dopamine, glutamate, opioid, and CRF signaling in the NAc, BLA, mPFC, and other prefrontal and subcortical regions (Bossert et al., 2007; Cruz et al., 2014; Millan and McNally, 2012; Prasad et al., 2020; Ritchie et al., 2024). However, most cellular and molecular mechanisms identified in addiction models, often derived from experimental designs involving chronic, experimenter-administered substance exposure without instrumental behavior or from reinstatement paradigms, have not been examined within translational R/R procedures. As a result, the applicability of these mechanisms to R/R-based interventions remains unclear. Nonetheless, emerging evidence implicates many transcriptional and epigenetic regulation, including dynamics in transcription factor binding, gene expression patterns, histone deacetylation and acetylation processes, chromatin remodeling, via interaction with other substances (i.e., cannabinoids), as a key process constraining or permitting relapse-related plasticity within brain reward networks (Mews et al., 2023, 2024; Martínez-Rivera et al., 2025; Walker et al., 2018; Blaze et al., 2024; Chisholm et al., 2025; Hurd et al., 2019; Romieu et al., 2011; Childs et al., 2024; Campbell et al., 2021a, 2021b; Wood et al., 2025; Hughes et al., 2024; Browne et al., 2025; Koijam et al., 2024; Picone et al., 2025; Teague and Nestler, 2022). Interestingly, additional processes involving Src-family kinase-mediated NR2B subunit-containing NMDAR activation in the dHipp, as well as orexin/hypocretin signaling at the orexin 1 receptor have been linked to memory processes tied to cocaine-associated renewal (Xie et al., 2013; Smith et al., 2010).

Importantly, these molecular mechanisms extend beyond neurons. Human and preclinical studies increasingly implicate glial processes, including neuroinflammation, myelination, and metabolic support, as regulators of relapse vulnerability, particularly under conditions of chronic drug exposure and stress (Holt et al., 2025). Although direct gene-therapy or gene-editing approaches for SUDs remain experimental (Yim et al., 2020), these findings identify clinically relevant non-neuronal targets, including inflammatory signaling, oligodendrocyte function, and metabolic regulation, that may complement circuit-focused interventions (Kohno et al., 2019; Virmani, 2023; Caspani et al., 2022; Yalçın et al., 2024; Bazzi et al., 2025).

#### 3.2.4 Future directions

Despite strong convergence across species, translating R/R mechanisms into clinical practice remains challenging. Human relapse unfolds within dynamic social and emotional environments that are difficult to fully model experimentally. Nevertheless, the alignment of behavioral, circuit, and molecular findings across rodents, NHP, and humans supports the relevance of context- and memory-based frameworks for understanding relapse. Integrating longitudinal human neuroimaging, real-world context monitoring, and circuit/molecular-informed behavioral interventions, as well as appropriate and consequential behavioral designs in animal research, will be critical for developing more durable, generalizable, and individualized treatments for SUDs. Importantly, sex differences in R/R processes remain insufficiently characterized, as much of the preclinical literature has historically relied on male subjects. Systematic inclusion of females and explicit examination of sex-specific circuit and molecular mechanisms will be essential for improving translational validity and ensuring that emerging interventions are effective across populations. Identifying biomarkers linked to contextual vulnerability, potentially through neuroimaging, behavioral phenotyping, behavior-based interventions, cellular and molecular techniques, may enable patient stratification and guide personalized interventions.
